# The many meanings of evidence: a comparative analysis of the forms and roles of evidence within three health policy processes in Cambodia

**DOI:** 10.1186/s12961-017-0260-2

**Published:** 2017-11-10

**Authors:** Helen Walls, Marco Liverani, Kannarath Chheng, Justin Parkhurst

**Affiliations:** 1 Faculty of Public Health and Policy, London School of Hygiene and Tropical Medicine, London, United Kingdom; 2 National Institute of Public Health, Phnom Penh, Cambodia; 3 Department of Social Policy, London School of Economics and Political Science, London, United Kingdom

## Abstract

**Background:**

Discussions within the health community routinely emphasise the importance of evidence in informing policy formulation and implementation. Much of the support for the evidence-based policy movement draws from concern that policy decisions are often based on inadequate engagement with high-quality evidence. In many such discussions, evidence is treated as differing only in quality, and assumed to improve decisions if it can only be used more. In contrast, political science scholars have described this as an overly simplistic view of the policy-making process, noting that research ‘use’ can mean a variety of things and relies on nuanced aspects of political systems. An approach more in recognition of how policy-making systems operate in practice can be to consider how institutions and ideas influence which pieces of evidence appear to be relevant for, and are used within, different policy processes.

**Methods:**

Drawing on in-depth interviews undertaken in 2015–2016 with key health sector stakeholders in Cambodia, we investigate the evidence perceived to be relevant to policy decisions for three contrasting health policy examples, namely tobacco control, HIV/AIDS and performance-based salary incentives. These cases allow us to examine the ways that policy-relevant evidence may differ given the framing of the issue and the broader institutional context in which evidence is considered.

**Results:**

The three health issues show few similarities in how pieces of evidence were used in various aspects of policy-making, despite all being discussed within a broad policy environment in which evidence-based policy-making is rhetorically championed. Instead, we find that evidence use can be better understood by mapping how these health policy issues differ in terms of the issue characteristics, and also in terms of the stakeholders structurally established as having a dominant influence for each issue. Both of these have important implications for evidence use. Contrasting concerns of key stakeholders meant that evidence related to differing issues could be understood in terms of how it was relevant to policy. The stakeholders involved, however, could further be seen to possess differing logics about how to go about achieving their various outcomes – logics that could further help explain the differences seen in evidence utilisation.

**Conclusion:**

A comparative approach reiterates that evidence is not a uniform concept for which more is obviously better, but rather illustrates how different constructions and pieces of evidence become relevant in relation to the features of specific health policy decisions. An institutional approach that considers the structural position of stakeholders with differing core goals or objectives, as well as their logics related to evidence utilisation, can further help to understand some of the complexities of evidence use in health policy-making.

## Background

It is widely agreed, including within the global health community, that data and evidence are essential to inform policy formulation and implementation [1–4]. However, the rhetoric of evidence-based policy – one based on the assumption that research is objective or unbiased and its uptake is a priori positive, with particular emphasis given to pieces of evidence classified at the top of so-called ‘hierarchies of evidence’ – has long been critiqued by social science scholars (c.f. [5–16]). For example, Weiss [17] has argued that research alone “*is almost never convincing or comprehensive enough to be the sole source of political advice*”, and “*there are always issues that research doesn’t cover*”. Increasingly, policy-studies scholars have explored aspects of the political system that may shape when, how and the types of evidence used within policy-making [7]. These can include both how political institutions (such as formal structures, and less formal rules and norms) [18] and how key ideas (including the way that issues are framed and understood) influence which types of evidence appear to be relevant for, and are used within, different policy processes [13, 19, 20].

However, as described by Oliver et al. [5], little empirical analysis has been undertaken of the processes or impact of evidence use in policy and the way that research and policy processes interact. This paper seeks to help address this gap through a comparative examination of the role that institutional and ideational factors play in shaping evidence use for three contrasting health policy decisions within a single country context. Specifically, this paper presents findings from research conducted in Cambodia, where the Ministry of Health (MoH), like many government departments in countries elsewhere [21–23], has explicitly embraced the overarching language of using ‘evidence-based’ approaches to health policy-making. One example of this endorsement is in the country’s second Health Strategic Plan (2008–2015), which defines priorities and goals for the entire health sector, highlighting the need “*to strengthen and invest in health information system and health research for evidence-based policymaking, planning, monitoring performance and evaluation*” [24]. In this context, our study aimed to examine and compare the ways evidence was discussed or used in three contrasting health policy areas, namely tobacco control, HIV/AIDS and performance-based financing (PBF). With regards to PBF, we focus on a widely-praised government midwifery incentive scheme (GMIS) that was introduced to increase deliveries at public health facilities.

Tobacco control represents a policy decision for which there is a long history of acknowledged corporate and governmental financial interests that have often attempted to influence how health-related evidence is used in regulatory policy-making [25–28]. HIV/AIDS, on the other hand, is an issue with strong donor and global interest, and which has seen policy ideas particularly shaped by global civil society movements and consensus [20, 29–31]. Finally, PBF tends to have much less external contestation or debate, but is largely seen as a more technical matter related to health economics, health service provision or health systems functioning [32, 33]. As such, these three examples provide useful ways to reflect on how the different institutional settings in which policy-making takes place may influence evidence use, including how interests and ideas of key actors within the differing institutional arrangements play out in relation to evidence utilisation.

## Methods

The paper draws on findings from semi-structured, in-depth interviews (IDIs) conducted in Cambodia in 2015 and 2016 with stakeholders from key health sector organisations, as well as a related documentary analysis. The interviews were undertaken as part of a wider research project examining political aspects of evidence use for health policy-making in multiple countries. In case-study countries, key informants were first asked questions about the systems and processes through which evidence was used to inform health policy broadly, followed by asking for multiple examples of recent health policy decisions that could be illustrative of different aspects of evidence use. In all countries, we subsequently investigated evidence use within tobacco control policy – given the importance of tobacco use for health in virtually every country context, as well as the existence of both a well-established evidence base and a global policy framework (i.e. WHO’s Global Framework Convention on Tobacco Control (FCTC)). After consultation with local stakeholders, we then selected additional country-specific health policy decisions of interest or importance to enable comparative analysis. As noted earlier, this approach led to the selection of three examples in Cambodia, namely tobacco control, HIV/AIDS and PBF.

Key participants were identified though purposive and snowball sampling strategies. In line with our approach, we first approached high-level policy-makers likely to be knowledgeable about major policy developments across the entire health sector, and who could thus provide a general overview of the systems and structures in place to use evidence and advise on the selection of case studies. Subsequently, a scoping review of relevant documents (i.e. published studies and grey literature in the public domain such as policy documents and reports) was conducted to collect background information on each policy issue. This was followed by identification of individuals who could comment further on the use of evidence to inform the selected policy decision. We endeavoured to conduct interviews with people who represented a diverse range of perspectives for the health decisions investigated. In total, 26 participants were interviewed, including both government representatives as well as individuals representing influential stakeholders in the policy process, particularly from aid providers, non-governmental organisations (NGOs), multi-lateral organisations and local research institutes.

IDI guidelines focused on broad topics, which were tailored to the different roles of informants and the specific expertise or insights they would bring; these were (1) perceptions about the policy process, including the role of different actors and contextual factors, (2) the nature and source of evidence that was used to inform the policy decision, (3) the way in which evidence was presented and evaluated, and (4) general views on institutional structures and practices of evidence use within the Cambodian health sector. Interviews were conducted face-to-face by the authors, recorded (if permission was given), and subsequently transcribed and coded into themes in an iterative process [34]. Citations from interviews and documents are included in the presentation of results to illustrate key points and emerging themes.

Consent was obtained at the initiation of each interview, with respondents given options on levels of anonymity desired. Ethical approval to undertake the study was provided by the London School of Hygiene and Tropical Medicine and research permission was obtained from the Cambodia National Ethical Committee for Health Research (n. 0120; 06/05/2014).

### Policy study perspectives

It is now reasonably well-established that national policy contexts can vary considerably with important implications for evidence use. Yet, even within a single country, the characteristics of evidence use for different health issues may also vary considerably. Previous work has made it clear that the political nature of policy-making means that there can be multiple competing interests and concerns at stake for any given policy decision – even within the health sector [7, 35, 36]. This indicates that multiple pieces of evidence may be relevant or considered in the policy process, depending on the differing concerns at stake, rather than any single piece or body of evidence. Thus, an important step in moving beyond an over-simplistic treatment of evidence use is to understand the differing interests of stakeholders holding varying power and influence over a given policy decision. Indeed, Cairney [7] notes that there can be such contestation at each step of the policy process – from defining the problem, to deciding which evidence to generate (or presumably which evidence to consider), to choosing solutions.

Scholars have thus begun to apply a range of theories and concepts from the policy sciences to help deepen our understanding of evidence use given these realities. Pearce [37], for instance, describes a ‘mistaken consensus’ that local climate policy can be based on emissions data, instead drawing out how ideas and arguments are also used, and needed, to construct local policy responses. This view is similar to that of Wellstead et al. [38], who argue that climate change adaptation science advocates are too narrowly functionalist in assuming that policies will change in response to feedback about climate change. Instead, they argue that understanding policy changes in this area requires looking not just at the specific problems climate science identifies, “*but also at the political and institutional factors that transform situations into problems and attempt to address them*” ([38], p. 13).

This shift away from thinking about policy problems as fixed, but instead to consider how issues become ‘problematised’, directly draws on the field of interpretive (or critical) policy studies, which considers the roles of rhetoric or discursive framing in shaping policy outcomes (c.f. [39–41]). It is not just climate science, however, which has seen such developments in analysis. In looking at health policy, Smith [13], for example, argues that it is the roles and interplay of ideas (and ideas about evidence) that can be critical to understand evidence use within differing health-related concerns.

The policy sciences have thus been increasingly applied to questions of evidence use in health policy-making and beyond. These perspectives allow consideration of the multiple interests and multiple bodies of evidence that are important to a policy decision, while further recognising the ways that institutional and ideational factors can lead to differing constructions of what evidence is seen to be appropriate to address any given interest in the first place – with institutional forms and ideas closely linked to the relative influence of different stakeholders in policy processes.

In this paper, we embrace this approach, applying ideas from new institutionalism to explore the competing or contrasting constructions of evidence use for a set of three differing health policy concerns in the setting of Cambodia. On the one hand, new institutionalism highlights not just the structures in place that shape decision-making processes and outcomes, but also the importance of rules and norms within organisations that guide actor behaviours or decisions [18, 42]. The approach also expands the focus of analysis beyond classic comparisons of state bureaucracies or legislative forms to consider the nature of institutionalised forces directing policy-relevant action across a much wider set of organisational forms, including non-state bodies, collections of stakeholders or contrasting elements within a government system.

Applying such an approach to the question of evidence use for health thus allows us to focus on multiple issues. First, we can consider the power or influence that different stakeholders have over policy processes based on their structural positions for a given policy issue – reflecting on how different bodies of evidence may be more or less relevant to given stakeholders with influence. However, this approach also allows exploration of the institutional logics which those stakeholders possess (c.f. [43, 44]) that further shape the use and understanding of policy-relevant evidence. In order to achieve these goals, we first provide an overview of the three policy areas addressed, followed by a description of the types of evidence seen to be applied or important in each case. This is then followed by our analytical section that applies this institutional and ideational lens to explore such questions.

## Results

### One country, three health policy issues

#### Tobacco control

Tobacco smoking became increasingly prevalent in Cambodia in the 1990s when the country was recovering from its civil war. At this time, there emerged the presence of many transnational tobacco companies in the country, the most prominent of which was British American Tobacco (BAT). The need for foreign investment and the lack of regulation of advertising at this time were explicitly recognised by BAT, who described Cambodia as “*an attractive and strategically important target*” [45]. A 1993 BAT industry plan, for example, acknowledged that awareness of the relationship between smoking and morbidity/mortality would increase in Cambodia through the activities of WHO, but estimated that “*the significant revenues generated by tobacco advertising* [for the government] *will, in the short term, delay anti-smoking initiatives until alternative forms of revenue are guaranteed*” [45]. BAT’s preferred option was reportedly to become a majority shareholder in a joint venture alongside local interests. Such an arrangement would presumably allow industry control of the composition of the company board of directors and significant influence over corporation activities, whilst also encouraging a local stake in the corporation’s success. BAT achieved this in 1995 [45].

According to Mackenzie et al. [45] there was also, at this time, owing to the lack of regulation, huge scope for tobacco-control advertising and promotional activities. Indeed, a 1994 survey of 12 main streets in the country’s capital Phnom Penh recorded that 49% of the advertising signs (8495 in total) were advertising tobacco products [46].

#### HIV/AIDS

HIV/AIDS in Cambodia has a very different history to tobacco smoking. In the mid-1990s, Cambodia had one of the fastest growing HIV prevalence rates in Southeast Asia, with injecting drug use and commercial sex driving HIV transmission [47]. Adult prevalence peaked at approximately 2.0% in 1998 [48]. However, since then, a number of prevention and treatment programmes have been introduced and the country’s prevalence had reduced to an estimated 0.7% in 2013 [49, 50].

The response has been divided into three phases. In phase I (1991–2000), a nationwide HIV prevention programme targeted brothel-based sex work, introduction of voluntary confidential counselling and testing, and home-based care, and peer support groups of people living with HIV emerged. Phase II (2001–2011) was characterised by expanding antiretroviral treatment (covering more than 80% of the population) and continuity of care, linking with other health services, accelerated prevention among key populations at higher risk (entertainment establishment-based sex workers, men who have sex with men, transgender persons, and people who inject drugs), engagement of health workers to deliver quality services, and strengthening health service delivery systems. Finally, phase 3 (2012–2020) aims to attain zero new infections by 2020 through sharpening responses to high-risk population groups, maximising access to community and facility-based testing and retention in prevention and care, and accelerating the transition from vertical approaches to linked/integrated approaches [50]. In recognition of the country’s success in halting and reversing the spread of HIV (relating to the United Nations Millennium Development Goal (MDG) 6), Cambodia was presented in 2010 with an MDG Award [51].

#### PBF and the case of the GMIS

The final health issue we explored in relation to the use of evidence was that of PBF, with specific discussion in the interviews about the role of evidence in supporting the GMIS. In many low- and middle-income countries, PBF is increasingly being used to redress particular aspects of health system underperformance, particularly the productivity and quality of healthcare providers. It involves offering incentives intended to redress underperformance, particularly high worker absenteeism, which is frequently observed in poorly funded public health systems with poor accountability [52]. Support for PBF has spread rapidly in many countries in recent years [52]. However, whilst there is considerable enthusiasm for PBF policies, according to a Cochrane Collaboration review [53] of pay-for-performance to improve the delivery of health interventions in low- and middle-income countries, the current evidence base is too weak to draw any general conclusions regarding effectiveness, with more robust and comprehensive studies being needed.

According to van der Poel et al. [52], Cambodia was the first documented case of a low-income country to experiment with PBF of public healthcare. Since 1999, a variety of health programme funding of districts and facilities in Cambodia have been contingent on performance targets or have directly linked revenues to services delivered. The main PBF programmes implemented have specified performance targets relating to child vaccination, antenatal care, delivery in a public facility, and birth spacing [52]. These funding arrangements have been intended to increase aspects of healthcare provision, and there has been considerable variation in the strength and conditions of the incentives offered [52].

The interviewees specifically identified the GMIS as a notable PBF policy, and described how the policy contributed to reducing Cambodia’s high maternal mortality ratio over recent years. The GMIS became operational nationwide in late 2007, following a joint *prakas* (directive) from the MoH and Ministry of Economy and Finance (MEF) to allocate government budget to the incentive payments [54]. The UNFPA was considered to be behind the policy change, for example, through supporting a High-Level Midwifery Forum in late 2005 that brought together representatives from several government departments. However, it was the prime minister who reportedly ‘gave the green light’ for the policy to go ahead. Other stakeholders were not thought to have had much direct influence over this decision, and the Cambodian Midwives Council, for example, was established after the implementation of the GMIS.

The GMIS aimed to boost facility deliveries by motivating skilled birth attendants (or trained health personnel) to promote deliveries in public health facilities. It did this by providing midwives (and other trained personnel) cash incentives based on the number of live births they attended in public health facilities, with US$ 15 for a live birth in a health centre and US$ 10 for a live birth in a referral hospital. The reason for the higher payment in a health centre than a hospital was to provide a stronger incentive for deliveries at health centres – the recommended facility for normal deliveries to be managed [55]. According to the MoH’s guidance, besides midwives, physicians and other trained health personnel can also receive these incentives when attending deliveries in public health facilities. Up to 30% of the incentives will be shared with other health personnel in the facility and eventually with other people, such as traditional birth attendants, who refer women to the facility for delivery [54]. The number of deliveries is reported monthly by health facilities through the routine health information system. Based on the number of reported deliveries, incentives are disbursed quarterly to the facilities through public financial disbursement channels [54].

### The nature of evidence used

In this section, we begin to describe and examine the reported differences in evidence use between the three policy areas. The evidence relating to each issue can be categorised in various ways, including by evidence topic (e.g. health, economic) or type (e.g. epidemiological, pilot study), which relate to the issue framing by key stakeholders, and the sources relied upon (e.g. global literature, national statistics, government survey). What is clear, however, is that no single uniform construction of policy-relevant evidence was seen across cases.

#### Tobacco control

Global evidence on tobacco harms were at this time considered well established, but local data on smoking rates were fairly limited. In the late 1990s, Cambodia had some small regional surveys of smoking prevalence, but it was not until 2005 that accurate nationwide prevalence data on tobacco use was available [56]. In spite of a lack of local data on smoking, in May 2004 Cambodia signed the FCTC, a global policy agreement that calls for a number of restrictions on tobacco advertising and promotion – restrictions which many global health authors present as ‘evidence based’ [57–59]. Many stakeholders interviewed noted the importance of the FCTC locally, as it dictated that the government could not engage with industry on developing tobacco control policy. However, implementation of the elements of the convention were described as only occurring slowly or in limited ways, which interviewees suggested was due to industry influence.

For example, one independent health sector consultant explained:

“*The tobacco industry and lobby is massively powerful here*.” (IDI-01, June 2014)

Another respondent, a senior civil servant in the MoH, explained:

“*We don’t know exactly why the law is very slow to be approved. Probably this is also due to lobbying of tobacco corporations, but we don’t have evidence to prove it*.” (IDI-10, August 2014)

This individual also noted that tobacco control did not appear to be a priority in the national health sector strategic plan.

The context of a deeply entrenched and powerful tobacco interest was also manifested in how respondents conceptualised the evidence that was relevant for moving tobacco control policies forward. For example, a number of civil servants interviewed stressed the need to counter other evidence that the tobacco industry uses to frame tobacco control in a way that suits industry interests. For example:

“*They* [tobacco corporations] *always complain that if we increase taxes, farmers will lose their job. So, you have to explain to the government that, if you increase taxes, the margin will not affect the industry. Also, we have to explain that farmers do not rely on one crop only, so reduced tobacco production will not significantly affect them*.” (IDI-06, June 2014)

“*Tobacco industry is powerful and has money. Some people are lobbied by tobacco corporations. They* [tobacco corporations] *have a lot of experience. They can approach friends or members of the family and get confidential information about policy making. Then, people that are lobbied create opposition at the inter-ministerial meetings. They often say that tobacco control will impact on the economy and farmers.*” (IDI-08, August 2014)

Respondents also emphasised the importance of making different evidence-based arguments to different actors. In particular, it was noted that the MEF needed different evidence from that required by the MoH regarding tobacco control to try to convince it to support policy action. As one civil servant explained:

“*You have to find a way to convince people… also because policy is multisectoral. It’s not that one minister decides. If you want to increase tax, this is not an issue of the Ministry of Health. We don’t have the power to do this. We can do a smoke-free policy, but tax is under the Ministry of Finance. So you have to work closely with the Ministry of Finance. In Cambodia, when you talk to the Ministry of Finance, first you have you prove to them they can make more money… The industry can say ‘oh if you increase taxes, you will lose revenue’. And you have to present evidence that increasing taxes is not a loss of revenue*.” (IDI-06, June 2014)

“*We explain to the government that an increase in tax does not change the overall volume of cigarettes that are sold in the market. The case of Thailand shows this. Why? Because smoking prevalence decreases, but population increases. Cambodia is the same. Smoking prevalence has gone from 49.6% to 42.6%, however the absolute number of smoking is always 2 million because population increased… and you have to tell the government these facts. So you have to do a lot of work with the government to prove this. And of course, the tobacco industry makes a lot of money. We cannot stop them*.” (IDI-06, June 2014)

This civil servant also explained how evidence from neighbouring countries was considered influential, as was the normative element of the FCTC.

“*Usually in Cambodia we present evidence or examples from ASEAN* [Association of South East Asian Nations] *countries. How is Vietnam doing? Thailand? Indonesia? Then, we also do international. But ASEAN is very important, also because we are approaching the ASEAN* [Economic] *Community in 2015, and member countries do not want to be left behind.*” (IDI-06, June 2014).

Overall, whilst many of the respondents spoke of the slow progress of tobacco control policy in Cambodia, the government has made substantial progress in tobacco control by banning the advertisement of tobacco products in 2011 as well as banning smoking in workplaces and public spaces in 2016 using sub-decrees. However, it was not until April 2015 that the Cambodian National Assembly passed the country’s first-ever law on tobacco control, which was ratified later the same month. The new law tackles tobacco from a variety of angles, including through import and sales restrictions, and bans on sales to minors and pregnant women [60].

#### HIV/AIDS

HIV/AIDS policy-making illustrates a radically different political context in which the utilisation of health policy-relevant evidence can be explored. UNAIDS has stated that Cambodia has “*used high-quality strategic information to inform a* [successful] *evidence-based response*” [61], and interviews stood in dramatic contrast to those with stakeholders advocating for greater tobacco control who expressed the need to develop or discuss evidence of financial impact (e.g. on farmers or the treasury) to justify policy action. Instead, in discussions of HIV/AIDS, whilst a variety of evidence types were clearly brought to bear, NGO and donor-organisation respondents discussed how it has often been epidemiological modelling and cost-effectiveness analyses (IDI-21, May 2016) – forms of evidence more typically advocated by public health actors for priority setting – that were seen as important evidence to guide policy. One of the respondents spoke about how this approach should be replicated in other areas of health policy-making:

“*I would say for HIV/AIDS it’s that way* [evidence use is more technical than in other areas of policy] *every time. I feel like that’s brilliant and the model of HIV/AIDS* [should] *be replicated to other diseases, for example, we still have a very high number of deaths among pregnant women, the baby, the infant. So why don’t they learn from the HIV/AIDS program*.” (IDI-21, May 2016)

When interviewees were asked about the use of evidence within particular policy developments, a range of evidence types were described in addition to the epidemiological modelling and cost-effectiveness studies mentioned above. Other relevant evidence was said to include pilot studies, used, for example, to inform the development of a community-based testing approach implemented in 2013, where the HIV testing is performed by lay counsellors – volunteers from population groups at higher risk of HIV infection. National prevalence estimates and international evidence were also evidence types that were frequently mentioned, particularly with international evidence from other Southeast Asian countries, and particularly Thailand. The importance of international evidence may also reflect the strong role of donors in HIV/AIDS policy-making in Cambodia.

“*Yes they* [policy-makers] *welcome* [overseas evidence] *in the HIV area. I don’t know about the other area. They welcome to learn the best practice from the region. This week one* [NGO] *staff member. He joined the field in Bangkok.*” (IDI-20, May 2016)

Epidemiological modelling of future prevalence scenarios has been considered key to informing policy with regards to the prioritisation and targeting of interventions, preparation of operational plans, budgets and resource mobilisation efforts [11, 12]. Further, cost-effectiveness analyses, such as modelling, have highlighted areas where technical efficiency might be improved. The National Centre for HIV/AIDS, Dermatology and STI (NCHADS) within Cambodia’s MoH, which is responsible for the health sector response to HIV and other sexually transmitted diseases, has led the analysis, in collaboration with relevant departments and centres of the MoH, the National AIDS Authority and other government institutions, as well as health service providers, NGOs, and other civil society organisations and development partners.

When asked about the reasons why HIV/AIDS policy-making stood out in terms of the use of what is more typically considered policy-relevant health evidence, respondents particularly spoke of the strong donor interest and support for HIV policy-making in Cambodia. They felt that this was key to driving the type of evidence being used in policy-making, and also the relatively well-functioning institutional entry points for such evidence, including the relevant technical working groups (TWGs) of the MoH. One NGO respondent explained:

“*Yes it’s different* [the policy-making process for HIV/AIDS compared to that for other health issues]. *I think this is because donor support, and I think the other thing is because of resource, donor support and resource. Resource, I would say financial resource and human capacity resource, let’s say for HIV/AIDS they have more educated* [staff] *and they adhere to plan, they adhere to target and they target evidence and I feel like the government take that approach very well, participatory approach, it’s very well, because it’s an emergency situation but it is also in the situation where funding is allowing so that’s why we feel like they are open*.” (IDI-21, May 2016)

However, there were downsides to a donor-driven approach also described, including in relation to siloed, non-integrated evidence gathering. When asked what could be improved, one interviewee explained:

“*Well I think it’s coordination. Because there’s basically the different donor programme, donors put all the evidence together. It sits on programmes that are… like some donors who are actually doing their own evidence, but not systematically led by the national programme and disseminated in a timely manner*.” (IDI-19, May 2016)

Interestingly, while HIV/AIDS policy-making has at times been seen as controversial or contested in some countries, related to the highly stigmatised nature of HIV transmission in some contexts [62, 63], we found little evidence of this in Cambodia. Whilst stigma and discrimination towards groups at higher risk of infection (sex workers, men who have sex with men, transgender persons and people who inject drugs) were noted, these were perceived to be relatively low compared to that in many countries elsewhere. Instead, one of the NGO respondents explained that, in Cambodia, policy-makers are relatively open – and increasingly so – to discussing these groups and considering evidence relating to these groups.

“*The policy-makers they are more open now… for HIV the policy-maker they are more open and learn from the* [experience of high-risk groups].” (IDI-20, May 2016)

The respondents from a key NGO also spoke at length about the effort made by the NGO to engage high-risk population groups in the policy-making process, particularly with regards to supporting representatives to speak at community meetings and at the MoH TWG meetings.

“[The NGO] *work to promote that involvement in the policy-making as well, not only* [the NGO] *but also civil society. But we try to involve the key representatives from each key population to enrol in the policy-making process… we use the number, we use the finding, we use the civil society. But also bring the key population to talk during the meeting is more powerful… So this is like an MSM* [men who have sex with men] *person or a sex worker, an entertainment worker could stand and speak about their challenges and the law enforcement people they listen to this. They’re part of the meeting. Everyone is part of the meeting… They also listen and sometimes they* [describe] *their challenges and you can see some improvement*.” (IDI-20, May 2016)

#### PBF and the case of the GMIS

In contrast to the evidence types seen as relevant for tobacco control and HIV/AIDS policy-making, when asked about evidence for policy-making in regard to PBF schemes generally, these schemes were described as reliant almost solely on evidence from pilot studies. Interview respondents spoke of various pilot schemes of PBF that had been run over the years in different districts and by different groups, often by NGOs, but also in workplaces of NCHADS. The perceived dominance of pilots as an evidence type is likely due to specific PBF policies being scaled up based on a pilot, but such schemes are likely also informed by evidence from health economics more broadly (and indeed, from basic microeconomics) that incentives can achieve outcomes [64]. When pushed for further examples of evidence use in the PBF area, some respondents mentioned evidence from the Demographic and Health Survey, and also international evidence as informing the use of such schemes; however, respondents did not provide specific examples of such evidence. Some mentioned the low payment of midwives and the lack of incentives for women to deliver in health facilities as evidence for needed change.

“*I don’t think anyone guided the government to design that policy. But it came clearly from many dialogues that the pay was not enough, that the arrangements did not encourage midwives to work in remote health centres, and did not encourage mothers to use health facilities*.” (IDI-11, June 2014)

“*A few years ago there was a policy to put one midwife in each health centre and the midwifery incentive… Hun Sen acted on this, and the policy was implemented immediately and very effectively… the trigger was the Demographic and Health Survey… it has quite a bit of impact, and there was a lot of pressure from the international community… it was a relatively ‘easy fix’, a simple solution*.” (IDI-12, June 2014)

However, the dominance of pilots as an evidence type fits with observations from van de Poel et al. [52], as stated above, of Cambodia’s pioneering role in experimenting with PBF of public healthcare, and also with one of our respondent’s description of Cambodia as “*a country of pilots*” (IDI-23, May 2016).

In contrast, however, there was also considerable discussion of the fact that, at times, policy directives come from high levels of government – within the MoH or as a decision made by the Prime Minister himself – and that, in such situations, evidence is perceived to be of limited importance. The GMIS was described as an example of this:

“*That* [the GMIS] *was an example of policy being changed by the government. It’s the government’s job, without any evidence*.” (IDI-15, May 2016)

“*You know, in the United States the evidence has, as far as can tell, no effect on congress. But what happens is they pass laws and then health and human services when they’re putting out the regulations or something, that’s where the evidence comes in. Here it’s more like everything at the congress level, even there is no, it doesn’t get more rational as it comes down through the MoH… People have very set ideas about things and those aren’t going to change no matter what evidence is put in front of them*.” (IDI-22, May 2016)

“*You can present the evidence and present it passionately and you can present it unanimously when you are heard, but there’s never really a proper policy dialogue. So saying well I could go and speak to the Ministry of Economy and Finance about that, we need extra money or we need to look at the budgets or perhaps we need to revise the* [pre-service] *training curriculum. You don’t get that*.” (IDI-15, May 2016)

“*We don’t even know who made it. There’s a re-writing of history that claims the MoH thought about it but there was no evidence. I was here right after it started and know many people here when it started. At the time no-one was claiming the MoH invented it, so it came out of the MEF, the Ministry of Economy and Finance. It was actually hugely successful*.” (IDI-22, May 2016)

Another respondent from a donor agency was unable to name evidence in support of the policy, and instead described how he saw the policy process for the GMIS.

“*I think in Cambodia evidence* [is not so important, rather the] *government want the community to deliver their baby at the health facility so the government just simply providing incentive to the health staff, community wide, so for delivery of one live birth delivery, they get US$15.00 this is the decision by the government and government budget and then see if they implemented that.*” (IDI-17, May 2016)

This perception of success appears to come from data showing increasing facility utilisation for delivery and falling mortality rates nationally after implementation of the programme. Since then, the percentage of deliveries in public health facilities has increased substantially, from 29% in 2006 to 57% in 2011, and the maternal mortality ratio has declined substantially from 473 per 100,000 live births in 2005 to 206 in 2010 [54]. Care, of course, is needed with interpretation of such evidence. A number of evaluations of PBF schemes such as the GMIS have been undertaken, and PBF policies have been credited with developments, including increasing utilisation by the poor, decreasing total family per capita health expenditure and encouraging better management [65], but drawing firm conclusions of causality can be problematic, particularly when such programmes have been implemented alongside other health sector reforms [66]. One respondent further commented that the quality of evaluations undertaken is often poor.

### Institutional features and logics of evidence use

The three policy areas presented show few similarities in how pieces of evidence were used in various aspects of policy-making, despite all being discussed or undertaken within a single MoH, and within a broad policy environment in which ‘evidence-based policy-making’ is rhetorically championed. In this section, however, we draw out some of the particular institutional and ideational features of the three health policy concerns that may help to explain these findings.

A starting point is to compare the institutionalised positions of influence of the key stakeholders in each case in order to reflect on how the relevance of particular evidence types fit with the interests of such stakeholders. This can then be followed by considering any contrasting institutional logics that similarly might help explain differences in evidence utilisation. Such logics could either be direct thinking about which evidence is relevant and why (such as how public health actors explicitly embrace hierarchies of evidence at times), or they may be related to the overarching goals or expectations of the actors involved, which subsequently shapes their uses of evidence (such as when particular types of evidence more naturally align with or fit broader goals).

In the case of tobacco control, the historical influence of the tobacco industry appears particularly relevant, and the nature of contestation for this issue appeared to be principally framed in terms of financial implications of tobacco control. Our respondents described the financial importance of tobacco for the agricultural sector and the national economy as the paramount concerns for any policy change. Tobacco control advocates are well aware of the need to present different evidence and frame the issue differently to address the concerns of the most influential stakeholders, with a particular distinction made between the health and economic evidence needed when speaking to policy-makers from the MoH and MEF, respectively. The need for taxation and other regulatory policy to be made outside the MoH illustrated how limited health-related evidence of tobacco harms could be in driving tobacco control policy forward on its own.

Evidence from neighbouring countries, and the FCTC, were said to be influential. However, even so, and despite considerable progress, policy change in line with these was described as particularly slow, as too was the development of what were considered more appropriate forms of evidence to guide tobacco policy from a public health perspective such as national smoking prevalence surveys. Despite global evidence on tobacco harms and increasing local data on smoking, it was the concerns regarding economic growth and the industry’s entrenched interests and lobbying documented in our study and elsewhere [45, 67, 68] that dominated the agenda. As such, this significantly appeared to slow down the translation of the FCTC into local tobacco control policies as well as the collection of, or action based on, forms of evidence typically seen as relevant to health promotion.

In contrast, the HIV/AIDS policy response in Cambodia developed in a rather different political context. With HIV/AIDS, there was no establishment of corporate interests and little obvious financial interest at stake for any major stakeholders. Instead, the issue may have achieved a relatively high level of priority for policy action in Cambodia due to the attention and resources this issue has been afforded by donor agencies and, possibly related to this, the well-functioning TWGs of the MoH for HIV policy-making, as described by our respondents. The institutionalisation of donor influence is, in fact, reflected within the country’s various strategic documents for guiding programme implementation, including the National Strategic Plan for HIV/AIDS 2011–2015 and Cambodia 3.0, a strategy developed by the country’s MoH to eliminate new HIV infections and congenital syphilis by 2020. Both are considered to be in line with the global targets and foci established by UNAIDS and the US PEPFAR programme (from which Cambodia is a recipient of funds) [49, 69]. However, these are highly technical global policy agencies that routinely promote, or operate based around, particular forms of evidence types, embracing international discourses of evidence-based policy-making. This may translate to the Cambodian context, particularly since there were no other strong interest groups to present alternative rhetoric or framing around the issue, and as such may have led to the use of evidence types in Cambodia more typically advocated by public health advocates in HIV/AIDS policy-making, as well as the observation by one NGO respondent that the National HIV/AIDS Centre is “*big on evidence*” (IDI-25, May 2016).

Indeed, while in many countries the issue of HIV testing has been subject to debate or controversy, particularly around issues of disclosure or confidentiality of people living with HIV, and the challenges associated with addressing HIV in often stigmatised groups such as men who have sex with men, transgender people and sex workers, these concerns were considered by our respondents to have been relatively unimportant in Cambodia (even if admittedly seen as sensitive). That HIV/AIDS is an issue with social connotations in Cambodia perhaps explains the use of narrative evidence – stories of the lived experiences of marginalised groups – to influence policy-making. However, the relatively low level of moral contestation for this issue in Cambodia was noted and used to explain the recent introduction of community-based rapid HIV testing (so-called ‘finger-prick testing’) by lay volunteer counsellors for high-risk population groups. Within this recent national policy, however, it was again evidence of effectiveness provided from a pilot study that could be seen to lead to policy change [70, 71].

Nevertheless, the importance of pilots was much more apparent, and described as the primary source of policy-relevant evidence for PBF. This was in contrast to tobacco policy appearing to require discussion of evidence of financial impact (linked to the influence of one set of interest groups), and HIV policy drawing particularly on epidemiological models and surveys (in line with norms and expectations of global health agencies). Again, we can look to the most influential stakeholders involved and their institutional logics to help explain the emphasis on pilots as a form of evidence in this case. Indeed, this helps to move away from the often criticised over-reliance on the idea that a single hierarchy of evidence can guide policy decisions, to instead consider the policy ‘appropriateness’ of particular forms of evidence [72–74].

Unlike the previous cases, the GMIS policy appears to have had few stakeholders outside the government itself. It was reportedly made from the highest levels of the government, with some interviewees speculating that it was driven by the Prime Minister’s office in particular in response to a feeling that some action must be taken to help achieve the maternal health MDG by 2015. The power and influence in this case appeared to be particularly hierarchical, with decision-making made through a planning and management orientation. Pilot studies, which examine feasibility of an intervention, are a first step in exploring novel interventions, novel applications of an intervention, or the feasibility of an intervention in a particular context when the effectiveness of that intervention may be context dependent [75]. For this reason, they are often considered important evidence of effectiveness as well as feasibility within a particular context and for complex interventions, on the premise that cultural appropriateness of interventions is important and can shape outcomes [76]. As such, pilot studies have been described previously as particularly applicable in health services and health systems research [77], but they also could be seen to be particularly relevant when a government has decided that wide-scale implementation of an intervention is a primary objective such as achieving an MDG target through reducing the high maternal mortality rate.

In Table 1 we present a summary of these findings. In particular, we highlight the stakeholders established to have dominant influence in each policy case, and their institutional interests and logics that help to explain the evidence said to be used by our interviewees.

**Table 1:** Characterisation of the institutional and ideational factors related to the health policy issue

Health policy area	Established dominant stakeholders	Institutional interests and logics	Nature of evidence used		
			Types	Topic	Source
Tobacco control	Industry, Ministry of Economy and Finance	Financial importance of tobacco for agriculture sector and national economy	- Regional surveys - Epidemiological - Economic	- Health - Health - Finance	- Local government, - Global repositories - Local sources
HIV/AIDS	International donors	Hierarchy of evaluation evidence; importance of achieving global targets	- Epidemiological - Economic - Pilot studies - Narrative	- Health - Finance - Health - Social	- Global, local sources - Local sources - Local sources - Citizens
Performance-based financing – Government Midwifery Incentive Scheme	Ministry of Health, Ministry of Economy and Finance, Prime Minister	Importance of achieving global targets; importance of achieving national implementation	- Pilot studies - Epidemiological	- Health - Health	- Local (government, NGO) - Local sources

## Discussion

Despite a common use of the language of evidence-based policy-making in the health sector, there are in fact many types of evidence that can speak to a variety of political concerns and mandates that play out in the policy process. Sometimes evidence use differs because the evidence needs vary in order to solve an agreed upon problem. At other times however, there may not be any agreement on the nature of the problem, and as such it is the features of power, interests and framing that serve as important drivers shaping which evidence informs policy considerations. An institutional approach can help to understand some of these dynamics. It can identify which stakeholders have established positions of power within differing health policy issues, linking their interests to the nature of evidence used. It can also help reflect on the institutional logics of these stakeholders which may further influence when or how evidence is utilised.

For tobacco, large and expensive national prevalence surveys were considered necessary evidence for intervention, even given considerable evidence from smaller studies of a high prevalence of smoking in the country, and the irrefutable global evidence linking tobacco to numerous diseases and mortality. However, such surveys were needed because of a demand for evidence that could speak to the dominant concerns of financial impact and the logic that evidence was needed to illustrate economic impact, rather than any public health logic of evidence to show medical harm to the population. The importance of the MEF is thus apparent in this case – illustrating both its dominant policy concern in terms of the economy, but also its logic of what forms of evidence are required to speak to that concern. Furthermore, it is of course critical to understand the historical influence and role of the tobacco industry in the country, which no doubt has played an important role in shifting the terms of tobacco policy to one of revenue.

In contrast, for the case of HIV/AIDS, the dominance of global donors in supporting this health issue, and the apparent limited contestation at a local level, appears to have led to the explicit embrace of epidemiological evidence that is widely held to be appropriate for HIV planning within the global health community. However, in the final case of PBF, it was the government that drove both the initiation and implementation of the policy response. This state-controlled process appeared to reflect a belief that national action must be taken to address an existing priority (in the form of a MDG). This, in turn, naturally led to a logic which saw relatively small studies focussed on implementation to be the most relevant to policy. Although it is worth noting that, in the case of the GMIS, some believed that evidence was not perceived as important at all due to the policy being driven by higher level political authorities. Indeed, some stakeholders simply referred to the GMIS policy when asked for a good example of the use of evidence, since it was national action based around the ‘evidence’ that maternal mortality rates were too high. This is a much simpler logic of evidence-informed policy-making, whereby evidence of a problem is seen to justify widespread action, in contrast to more traditional health sector descriptions of evidence use being concerned with the effectiveness of interventions or possible alternative priorities or approaches.

Policy studies scholars would not be surprised that powerful stakeholders (or ‘vested interests’) end up shaping the understanding of evidence, or which pieces of evidence are championed as relevant for policy-making. This is perhaps most clearly illustrated in how evidence has been presented or selected by the tobacco industry in relation to policy-making debates (c.f. [27, 28, 78, 79]). For example, tobacco industry-funded studies have been shown to have misrepresented the association between second-hand smoke and cardiovascular diseases [27], or the evidence in support of standardised packaging of tobacco products [79]. Tobacco interests have also emphasised evidence in support of the economic contributions of their product, whilst questioning the evidence suggesting that policy interventions are needed to protect health [78]. HIV/AIDS, on the other hand, touches on issues of gender and sexuality, drug use and sex work, which often leads to it being seen as a highly morally contested issue. However, these did not appear particularly relevant in Cambodia, serving as a reminder that we cannot assume a health topic will necessarily exhibit the same political characteristics in different settings, even if such features are commonplace in other cases.

## Conclusions

In the three contrasting case studies of evidence use in health policy-making examined in this study, evidence types – and their framing – were found to differ greatly, despite them taking place in the same country setting. The findings reiterate past authors’ understandings that ‘evidence’ is not a uniform concept for which more is obviously better, or where a single model of ‘evidence-based policy-making’ can prevail, but rather that different constructions and pieces of evidence become relevant given the politics involved in policy decisions, the nature of institutions involved, and the framing and conceptualisations of the issues themselves. Our comparative analysis helps to begin to trace out themes in linkages between the nature of contestation of health issues, the interests of established dominant stakeholders, and the logics by which those stakeholders operate – all of which work to shape which evidence is utilised or seen as policy relevant to inform health decisions. Whilst considerable further empirical research is needed in this area, this more nuanced understanding of evidence use may be of relevance to health policy-makers and others considering how to improve the role of evidence in health policy-making.

## Abbreviations

BAT: British American Tobacco
FCTC: Framework Convention on Tobacco Control
GMIS: government midwifery incentive scheme
IDI: in-depth interview
MDG: Millennium Development Goal
MEF: Ministry of Economy and Finance
MoH: Ministry of Health
NCHADS: National Centre for HIV/AIDS, Dermatology and STI
NGO: non-governmental organisation
PBF: performance-based financing
TWG: technical working group
